# Antibodies to the Core Proteins of Nairobi Sheep Disease Virus/Ganjam Virus Reveal Details of the Distribution of the Proteins in Infected Cells and Tissues

**DOI:** 10.1371/journal.pone.0124966

**Published:** 2015-04-23

**Authors:** Lidia Lasecka, Abdelghani Bin-Tarif, Anne Bridgen, Nicholas Juleff, Ryan A. Waters, Michael D. Baron

**Affiliations:** 1 The Pirbright Institute, Ash Road, Pirbright, Woking, Surrey GU24 0NF, United Kingdom; 2 School of Biomedical Sciences, University of Ulster, Cromore Road, Coleraine BT52 1SA, Northern Ireland, United Kingdom; Division of Clinical Research, UNITED STATES

## Abstract

Nairobi sheep disease virus (NSDV; also called Ganjam virus in India) is a bunyavirus of the genus *Nairovirus*. It causes a haemorrhagic gastroenteritis in sheep and goats with mortality up to 90%. The virus is closely related to the human pathogen Crimean-Congo haemorrhagic fever virus (CCHFV). Little is currently known about the biology of NSDV. We have generated specific antibodies against the virus nucleocapsid protein (N) and polymerase (L) and used these to characterise NSDV in infected cells and to study its distribution during infection in a natural host. Due to its large size and the presence of a papain-like protease (the OTU-like domain) it has been suggested that the L protein of nairoviruses undergoes an autoproteolytic cleavage into polymerase and one or more accessory proteins. Specific antibodies which recognise either the N-terminus or the C-terminus of the NSDV L protein showed no evidence of L protein cleavage in NSDV-infected cells. Using the specific anti-N and anti-L antibodies, it was found that these viral proteins do not fully colocalise in infected cells; the N protein accumulated near the Golgi at early stages of infection while the L protein was distributed throughout the cytoplasm, further supporting the multifunctional nature of the L protein. These antibodies also allowed us to gain information about the organs and cell types targeted by the virus *in vivo*. We could detect NSDV in cryosections prepared from various tissues collected post-mortem from experimentally inoculated animals; the virus was found in the mucosal lining of the small and large intestine, in the lungs, and in mesenteric lymph nodes (MLN), where NSDV appeared to target monocytes and/or macrophages.

## Introduction

The genus *Nairovirus*, of the family *Bunyaviridae*, includes viruses known to be pathogenic in humans or livestock, as well as viruses whose mammalian and arthropod hosts are still to be determined. Nairoviruses are classified into seven serogroups [1–3], of which the most important are the Crimean-Congo haemorrhagic fever (CCHF) group, which includes the human pathogen Crimean-Congo haemorrhagic fever virus (CCHFV), and the Nairobi sheep disease (NSD) group, to which belong Nairobi sheep disease virus (NSDV) and Dugbe virus (DUGV).

NSDV causes an acute haemorrhagic gastroenteritis in sheep and goats with a mortality rate up to 90% in a susceptible population [4–7]; the virus is endemic in East and Central Africa, where it was firstly discovered in 1910 [6, 8–12]. An Asian virus causing the same disease is called Ganjam virus (GV) (reviewed in [13]); based on genetic and serological studies, it has been identified as a variant of NSDV [12, 14].

Neither virus is contagious; in natural infection the virus is transmitted by ixodid ticks [6, 9, 15–17]. Sheep and goats are the only known mammalian reservoir of NSDV [8, 9]; other livestock are refractory to disease even when deliberately infected [6]. In most cases, infected animals which recover from disease remain refractory to subsequent infections for several years or even the lifetime of the animal [6].

While post-mortem examination of infected animals revealed inflammation and haemorrhage in the gastro-intestinal track, and enlarged spleen and lymph nodes [6, 18], the target cells of the virus remain unknown.

NSDV is closely related to Crimean-Congo haemorrhagic fever virus (CCHFV) which causes haemorrhagic fever in humans with an average mortality rate of 30% (reviewed in [19, 20]. After Dengue virus (DENV; genus *Flavivirus*, family *Flaviridae*), CCHFV is the second most widespread of the arboviruses pathogenic to humans, with cases reported in sub-Saharan Africa, the Indian subcontinent, the Middle East and Europe [21–29].

Nairoviruses are enveloped viruses with a diameter of approximately 100 nm [30–32]. The genome of nairoviruses consists of three single stranded negative-sense RNA segments [2, 33, 34] which are encapsidated by the viral nucleocapsid protein (N protein) forming, together with the viral RNA, the ribonucleoprotein (RNP) [2, 35–37]. The three RNA segments are called small (S), medium (M) and large (L) and encode respectively the N protein [2, 37, 38], the viral structural (i.e. Gn and Gc) and non-structural glycoproteins [2, 39–42], and the RNA-dependent RNA polymerase (RdRp) (L protein) [43–46].

Nairoviruses are thought to enter cells by clathrin-mediated endocytosis [47, 48]; after release of RNPs from the endosome, replication takes place in the cytoplasm [31, 49]. Once viral RNAs and proteins are synthesised assembly of the viral particles occurs most probably at the Golgi membranes [50]. Newly assembled virions bud into the Golgi and are transported, probably through the secretory pathway, to exit sites at the plasma membrane [31, 32, 49].

As for all bunyaviruses, the N protein is the most abundant viral protein in the nairovirus virion and in the infected cell, as the N protein encapsidates newly synthesised viral genomic RNA (vRNA) and complementary RNA (cRNA) [2, 34, 51–53]. The N protein of nairoviruses, at approximately 53 kDa, is almost twice in size of the N protein of viruses from other genera of the family *Bunyaviridae*, with the exception of hantaviruses [2, 54]. The L protein is also much larger than those of other bunyaviruses; the L segment is approximately 12 kb long, encoding a protein of approximately 450 kDa, almost twice as long as the L proteins of most other bunyaviruses, the exception being tospoviruses, the L protein of which is approximately 320 kDa protein [43, 44, 55, 56]. The L proteins of all bunyaviruses contains the viral RdRp activity [43]; the L protein of nairoviruses also contains regions of unidentified function at both the N- and C-terminus [56]. Notably, the L protein of nairoviruses contains additional domains upstream of the RdRp motifs (e.g. topoisomerase-like domain and zinc-finger domain), of which the ovarian-tumour (OTU)-like protease domain is the most studied [44, 56–61]. The OTU domain belongs to a larger papain-like cysteine protease family, members of which are found in other viruses, in *Saccharomyces cerevisiae*, in *Drosophila melanogaster* and in mammalian cells [62, 63]. Most of the studies on the nairovirus OTU have concentrated on its activity as a protein modifying enzyme, de-conjugating ubiquitin (Ub) and a Ub-like molecule from a wide variety of protein targets, which allows nairoviruses to avoid induction and action of type I and II interferon (IFN) [57–61, 64].

The OTU-like domain has been also found in other viral polyproteins, of which some undergo autoproteolytic cleave to generate multiple proteins. For instance, a non-structural replication-associated protein p223 (223 kDa) containing the RNA polymerase domain, encoded by ORF1 of blueberry scorch virus (BlScV) of the genus *Carlavirus* (family *Betaflexiviridae*) is autoproteolytically cleaved into two products of approximately 160 kDa and 60 kDa [65]. The nsp2 protein encoded by ORF1a of the arteriviruses (family *Arteriviridae*) equine arteritis virus (EAV) and porcine reproductive and respiratory syndrome virus (PRRSV) autoproteolytically cleaves at the nsp2/3 junction of the replicase polyprotein, which results in the separation of two non-structural proteins nsp2 and nsp3 [61, 66]. Because of the large size of the nairovirus L protein and presence of the OTU-domain, several authors have suggested that the L protein of nairoviruses may also be autoproteolytically cleaved into an active RNA polymerase and protein(s) with additional function(s) [44, 52, 56]. Although no specific cleavage sites have been described for OTU-like cysteine proteases, several potential cysteine protease-like cleavage sites have been identified in the L protein sequence of CCHFV [56].

Over all, the proteins of nairoviruses are still poorly characterised, with most of our knowledge coming from recent studies on CCHFV, which are limited by the difficulties of working with the virus. We generated specific antibodies to the N protein and the L protein of NSDV to characterise these proteins in NSDV-infected cells. To investigate whether the L protein of NSDV is cleaved in infected cells, separate antibodies against the N-terminus and the C-terminus of the L protein were prepared. We have used these anti-NSDV antibodies to characterise the distribution of the viral proteins in infected cells and the tropism of the virus in animals which were experimentally infected with a pathogenic NSDV isolate.

## Materials and Methods

### Cells and viruses

Cell lines and cell culture used in this work was as previously described [18, 67]. PO (sheep kidney epithelium) cells [68] were obtained from the Collection of Cell Lines in Veterinary Medicine (CCLV), The Friedrich Loeffler Institute, Riems, Germany. The highly tissue culture passaged NSDV isolate from Uganda (NSDVu), and the pathogenic isolate (NSDVi) (IG619,TVPII236), were also as previously described [18, 67].

### Virus infection of eukaryotic cells

Vero (African green monkey kidney) cells or PO cells were plated at an initial seeding density of 5 x10^4^ cells/well in 12-well plates. On the next day, cells were infected with NSDVi or NSDVu at a multiplicity of infection (MOI), calculated from 50% tissue culture infectious dose (TCID_50_), as indicated for each experiment. The virus inoculum was removed after one hour, cells were washed once with phosphate buffered saline (PBS) and fresh medium was added. The infected cells were further incubated for the times indicated for each individual experiment.

### Immunofluorescence

Vero and PO cells were cultured on 18 mm diameter cover slips prior to infection as above. At the indicated times, cells were fixed with 4% paraformaldehyde (PFA), washed with PBS and then permeabilised with ice-cold methanol for 5 min. The fixed and permeabilised cells were blocked with 0.2% porcine gelatine for at least 30 min before antibody staining as described [67].

Sections were viewed and images taken using sequential scanning on a DMIRE2 Leica CLSM TCS-SP2 confocal laser scanning microscope. The TIFF images obtained were resized and overlays were prepared with Adobe Photoshop.

### Cryosections

Samples were taken from animals VU19 and VU20 infected with the pathogenic NSDV isolate (NSDVi) in a study previously described [18], or from healthy animals that were not subject to any experimental procedures. Tissue samples collected at necropsy were snap frozen in Tissue-Tek O.C.T Compound (Sakuru, UK) and stored at -80°C until processing. 8 μm thick cryosections were prepared (Leica CM1900 Cryostat, UK) on Superfrost Plus microscope slides (VWR International, UK). Sections were fixed in 3% PFA for 15 min, washed with PBS and proteins partially denatured in cold (-20°C) methanol for 5 min. Sections were blocked in 10% normal goat serum for 30 min and then labelled with mouse or rabbit primary antibodies as indicated in the text for 45 min at room temperature. After washing four times in PBS, the sections were stained with secondary (fluorescent) antibodies as indicated in the text. Sections were washed a further four times, including 4',6-diamidino-2-phenylindole (DAPI) in the 2^nd^ last wash, before mounting in Fluorescent Mounting Medium (DAKO). Confocal images were obtained, TIFF files resized and overlays prepared as described above.

### Antibodies

Antibodies were raised against bacterially expressed fusion proteins consisting of the amino-terminal 167 amino acids of the NSDV N protein or the amino-terminal 169 amino acids of the NSDV L protein, fused to a 6xHistidine tag in vector pET23d, or the 521 carboxy-terminal amino acids of the NSDV L protein fused downstream of glutathione-S-transferase (GST) in vector pGEX6p. The fusion proteins were expressed in *E*. *coli* BL21(DE3)pLysS (Promega). At all temperatures and growth conditions tested the expressed proteins were insoluble. The insoluble inclusion bodies were washed and resuspended in PBS. Protein concentration was determined using urea-dissolved protein and the Coomassie (Bradford) Protein Assay Kit (Pierce). Rabbit antisera to the bacteria-expressed proteins were prepared by Cambridge Research Biochemicals. Affinity-purified antibodies were prepared from the positive antisera essentially as described by Olmsted [69]. Mouse monoclonal antibodies used in cryosections staining were: anti-cytokeratin (clone KS 1–8, AbD Serotec), anti-collagen IV (clone CIV 22, DAKO), anti-L1/calprotectin (clone MAC387, AbD Serotec), anti-CD31 (clone CO.3E1D4, AbD Serotec). Mouse monoclonal antibodies recognising sheep CD2 and CD45 were gifts from Dr C. Mackay, Basel Institute for Immunology, Basel, Switzerland. Alexafluor-488 and Alexafluor-568 conjugated anti-rabbit IgG and anti-mouse IgG antibodies were obtained from Life Technologies.

### Zenon labelling

To study the simultaneous localisation of viral proteins in a single cell, using two different rabbit antisera, Zenon Rabbit IgG Labelling Kit (Life Technologies) was used to independently label antibodies. The Zenon reagent to antibody molar ratio was determined experimentally and is indicated for each individual experiment. Cover slips containing infected cells were sequentially incubated with the first rabbit antiserum or affinity purified antibody for one hour at room temperature, washed four times with PBS and labelled with Zenon Alexa Fluor 594 rabbit IgG labelling reagent, containing fluorescently labelled Fab fragments, in a total volume of 20 μl, for 7 min. Unattached Fab fragments were removed by four washes with PBS. The second antiserum or affinity-purified antibody was prepared as a complex before incubating with the fixed cells: rabbit antiserum or purified antibody at the appropriate dilution was incubated with Zenon Alexa Fluor 488 rabbit IgG labelling reagent for 7 min; free Fab fragments were neutralised with Zenon blocking reagent (at an equal volume to Zenon Alexa Fluor 488 rabbit IgG labelling reagent) for 5 min at room temperature. The volume of this staining complex was made up to 21 μl with 0.2% porcine gelatine and the cells were incubated with this IgG-Fab complex for 1 h at room temperature. The excess of the antibodies and Fab fragments was removed by washing the cells four times with PBS. The cells were then fixed again with 4% PFA for 10 min to stabilise the Zenon-labelled antibodies attached to their target proteins.

### Analysis of confocal images using Imaris software

For detailed quantitative analysis of the distribution of the viral proteins by confocal microscopy, Imaris software was used. For each cell being analysed, a series of confocal images composed of 8–14 focal plane slices (taken through the thickness of the cell) were generated using sequential scanning with the confocal laser microscope. These image stacks were analysed for colocalisation of viral proteins using the ImarisColoc function of the Imaris x64 version 7.4.2 software, applying automatic threshold determination as described [70]. Image stacks obtained from infected cells stained with anti-L C-terminus antibodies separately coupled with both Alexa Fluor 488 and Alexa Fluor 594 were used as a control for perfect colocalisation and to normalise colocalisation data.

### Immunoblotting

At the indicated times, infected cells were harvested and lysed with 100 μl of 1x SDS sample buffer (New England Biolabs). SDS-PAGE and Western blots were carried out as previously described [71]. For the detection of proteins larger than 200 kDa, the Western blot transfer was performed using a TransBlot SD Semi Dry Electrophoretic Transfer Cell (Bio-Rad) and Bjerrum and Schafer-Nielsen transfer buffer (48 mM Tris, 39 mM glycine, 37.5 mg/L SDS, 20% methanol, pH 9.2) [72]. The Western blots were exposed using Kodak Image Station 4000R Digital Imaging System operated by Kodak Molecular Imaging Software (MI).

### Statistical analysis

The significance of differences observed in colocalisation of the N and L proteins at different time points post infection was analysed using the General Linear Model form of ANOVA, as implemented in Minitab 16, with hours post infection (hpi) as fixed factor and cell as a random factor. The Tukey Simultaneous Test was used to test the significance of differences observed between the groupings.

### Ethics statement

All experimental protocols and procedures for acquisition of post-mortem tissue samples were reviewed and approved by the local Pirbright Institute ethical review process (The Pirbright Institute Animal Welfare and Ethics Committee) and were subject to the provisions of the Animals (Scientific Procedures) Act 1986.

## Results

### Generation of specific anti-N and anti-L protein antibodies

**Fig 1:**
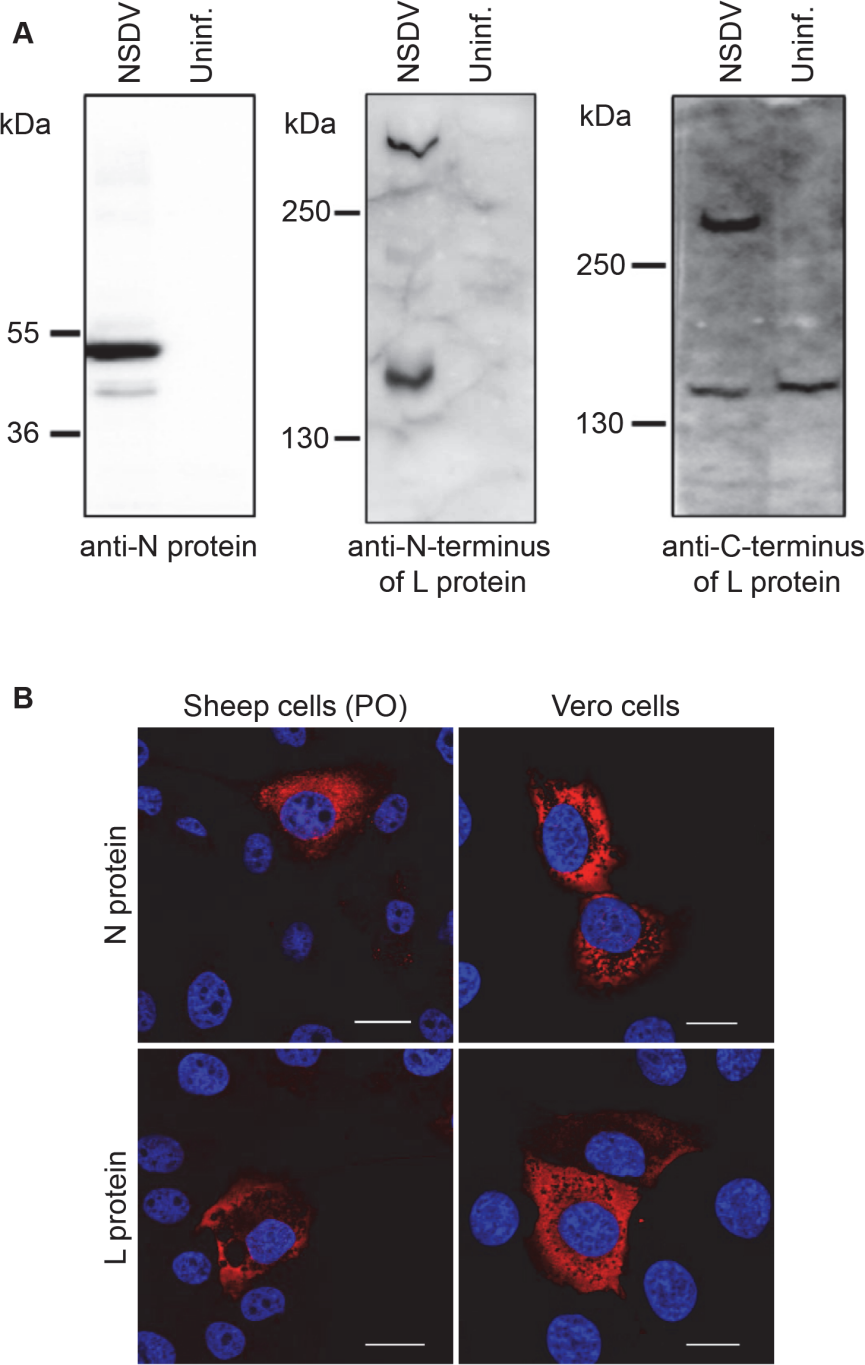
Characterisation of NSDV core proteins in infected cells. **(A)** Vero cells were infected with the NSDVi isolate at a MOI of 5 TCID_50_ (NSDV) or left uninfected (uninf.). After 16 h, cells were harvested, lysed in sample buffer and proteins separated by SDS-PAGE; proteins were detected by Western blot using sera raised against the NSDV N protein, the C-terminus of the L protein or the N-terminus of the L protein, as indicated. **(B)** Sheep kidney epithelial cells (PO) or Vero cells were infected with the NSDVi isolate at a MOI of 0.3 TCID_50_. After 16 h, cells were fixed using 4% PFA followed by ice cold methanol, and immunolabelled using sera raised against the NSDV N- or the C-terminus of the L protein followed by AlexaFluor-568 goat anti-rabbit IgG (red). DAPI was used as a counterstain (blue). Bars indicate 20 μm.

The antisera were raised in rabbits to bacterially expressed proteins representing the amino-termini of the N and L proteins, and the C-terminus of the L protein. The antisera recognised specific proteins of the expected sizes for the N or the L protein in infected cells (Fig 1A) and showed similar, though not identical, specific reticular staining patterns in NSDV-infected sheep epithelial cells (PO cells) and Vero cells (Fig 1B and S1 Fig).

### The L protein of NSDV appears to remain uncleaved in infected cells

**Fig 2:**
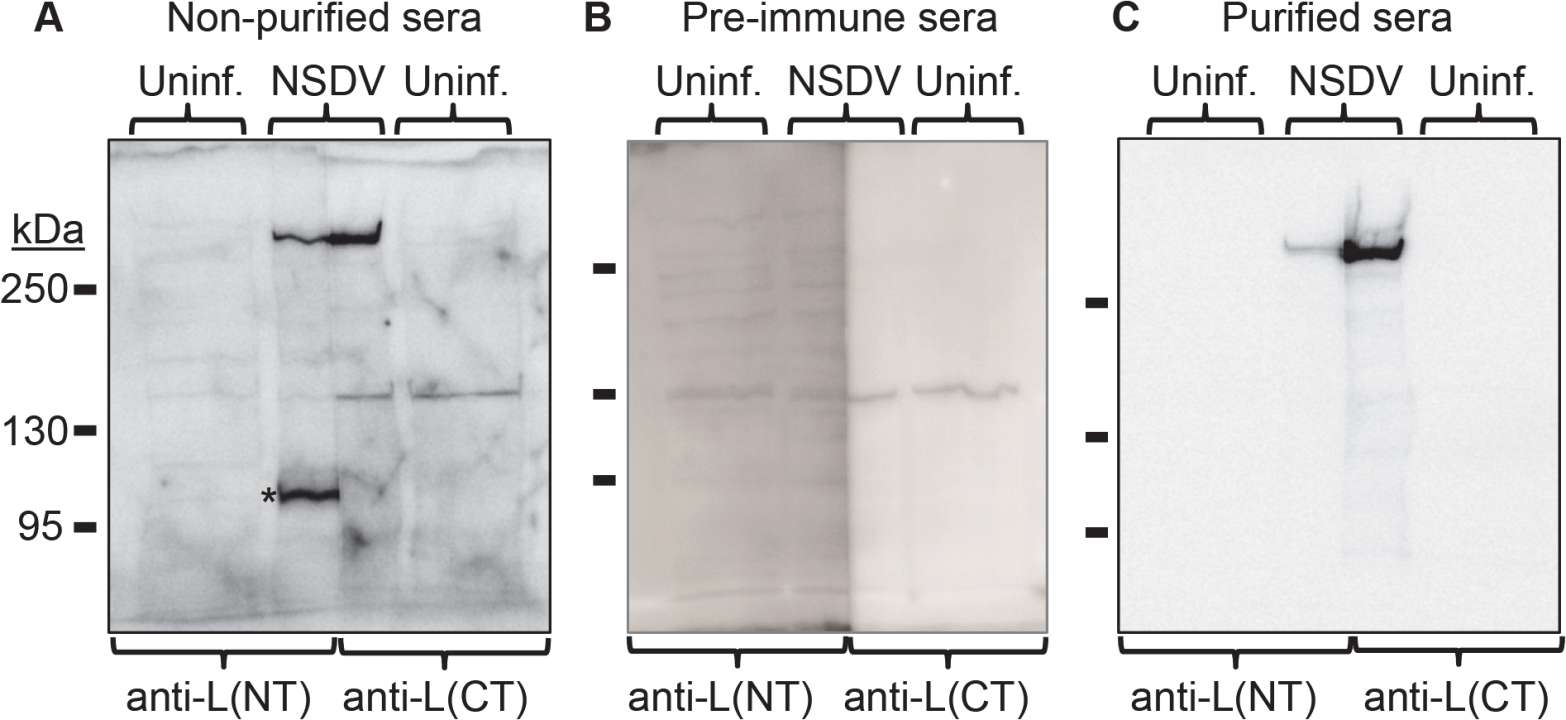
Detection of the full length L protein in NSDV-infected cells. Vero cells were infected with the NSDVi isolate at a MOI of 6 TCID_50_ or left uninfected (uninf.). After 16 h, infected and uninfected cells were harvested using SDS Sample Buffer containing protease inhibitors, and proteins were separated on 5% acrylamide SDS-PAGE gels and transferred onto polyvinylidene difluoride (PVDF) membrane. The membrane was cut vertically along the middle of the track containing proteins from infected cell lysate. Blots were then incubated with the indicated antiserum or purified antibodies before developing with HRP-anti-rabbit IgG. **(A)** Filters were incubated with diluted antiserum raised against the N-terminus of the L protein (anti-L(NT)) or the C-terminus of the L protein (anti-L(CT)). **(B)** Filters were incubated with the pre-immune sera corresponding to the sera used in A. **(C)** Membranes were incubated with affinity purified antibodies extracted from the sera used in A. For all immunoblots the migration position of protein size markers are indicated. The star (*) indicates a non-L protein peptide labelled by crude antiserum but not by affinity-purified antibody.

Because of the low percentage gel required to get efficient transfer of the full sized L protein (450 kDa), the resultant bands were frequently slightly distorted (Fig 1); it was therefore difficult to be sure that proteins detected with different antibodies, even if originally run in adjacent tracks, were actually of the same size. To be sure that the antibodies against the N-terminus and the C-terminus of the L protein recognise the same single protein, lysates from infected cells were applied to a single, wide well of an SDS-PAGE gel and, after the proteins were separated and transferred to Western blot membrane, the wide track was bisected vertically and each half was stained with anti-L N-terminus or anti-L C-terminus antiserum. A single band, corresponding to the size of the intact L protein, was detected in infected cells by both sera (Fig 2A). In addition, other proteins of various sizes (100–150 kDa) were also detected by one or the other antiserum, and in some cases these proteins were not detected in uninfected cells. In order to be sure that we were detecting only those proteins recognised by antibodies elicited by the original fusion proteins, and not some other component of the serum IgG, we affinity-purified the specific antibodies recognising the viral proteins. This was done using the method of Olmsted [69] with the original fusion proteins, purified by SDS-PAGE, as the affinity matrix. Using these affinity-purified antibodies, we observed only a single infection-specific protein of size corresponding to that expected for the full-length L protein (Fig 2C). No smaller protein which could represent a specific cleavage product containing the N- or C-terminus of the L protein was recognised by the affinity-purified antibodies, even when using high percentage gels (not shown), although we did detect small amounts of non-specific breakdown products, the amounts of which depended on the age of the original sample. These data show that all the detectable L protein in infected cells is present as the full-length protein, and argue strongly against autoproteolytic cleavage which would result in the N- and C-terminus being in different proteins.

**Fig 3:**
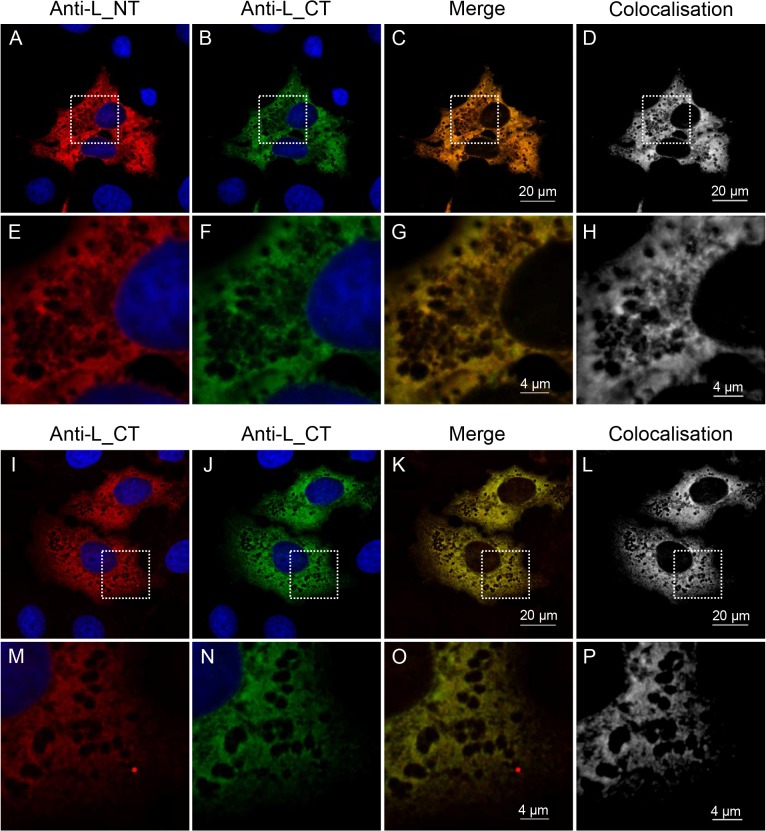
Colocalisation of the N-terminal and the C-terminal part of the L protein in NSDV-infected cells. Vero cells were infected with the NSDVi isolate at a MOI of 1 TCID_50_. After 16 h, cells were fixed using 4% PFA followed by ice cold methanol. **(A-H)**: Cells were stained sequentially with affinity-purified antibodies directed against the N-terminus of the L protein, Zenon AlexaFluor 488 (green) rabbit IgG labelling reagent (Fab:antibody ratio 5.6), and pre-made complex of affinity purified antibodies against the C-terminus of the L protein with Zenon AlexaFluor 594 (red) rabbit IgG labelling reagent (Fab:antibody ratio 3). **(I-P)**: Cells were stained sequentially with affinity purified anti-L(C-terminus) antibodies, Zenon Alexa Fluor 488 (green) rabbit IgG labelling reagent (Fab:antibody ratio 3.6), and then again with pre-made complex of affinity purified anti-L(C-terminus) antibodies with Zenon Alexa Fluor 594 (red) rabbit IgG labelling reagent (Fab:antibody ratio 3). Nuclei were counterstained using DAPI (blue). Representative focal planes from Z-stack series are shown. The ImarisColoc function of the Imaris x64 version 7.4.2 software was used to generate a colocalisation channel (D, H, L, P) from each 3D image, each of which was generated from eight focal planes through the thickness of an infected cell. Dashed white boxes in A-D and I-L indicate the area enlarged to show in E-H and M-P. Scale bars are shown.

These findings were confirmed by confocal immunofluorescence microscopy, which was used to study the specific localisation of the L protein N- and C- termini in infected cells. In order to use two rabbit antibodies in the same cell we employed the Zenon Rabbit IgG Labelling Kit. Using this system, affinity-purified anti-L(N-terminus) and anti-L(C-terminus) antibodies were directly coupled with two different fluorescently labelled Fab fragments, allowing for independent visualisation of these antibodies in the same cell. The absence of cross-labelling with the Zenon reagents was verified by control experiments in which one or other specific antiserum was replaced with the corresponding pre-immune serum while staining fixed infected cells (S2 Fig). Cells which were incubated with anti-L antibody/Zenon AlexaFluor 594 (red) and then with the pre-immune serum corresponding to the anti-N antibody coupled to the Zenon AlexaFluor 488 (green) showed only red fluorescence, while cells which were incubated with the pre-immune serum corresponding to the anti-L antibody/Zenon AlexaFluor 594 and then with anti-N antibody/Zenon AlexaFluor 488 showed only green fluorescence (S2 Fig). These data show that, under the conditions of our studies, there was no transfer of Zenon label between rabbit IgGs in/on the labelled cells, and also that the Zenon labels did not bind to cellular or viral proteins. Using this system, we labelled infected cells with the two types of anti-L antibodies (antibodies against the N- or the C-terminus of the L protein), and took series of images of different focal planes (Z-stacks) through the thickness of infected cells using sequential scanning on a confocal laser scanning microscope. The colocalisation detected using these anti-L antibodies (Fig 3A–3H) was identical to the colocalisation observed for a positive control where infected cells were sequentially stained using a single antiserum (anti-L(C-terminus)) directly and independently labelled with both Zenon reagents (594 and 488) (Fig 3I–3P), which was taken as a measure of 100% colocalisation due to targeting of the same epitope by both fluorophores. This control also allowed for normalisation for the effects of unequal bleaching of the 594 and 488 fluorophores, which could result in differential intensity of the two fluorophores in a single voxel even if labelling the same antibody.

**Fig 4:**
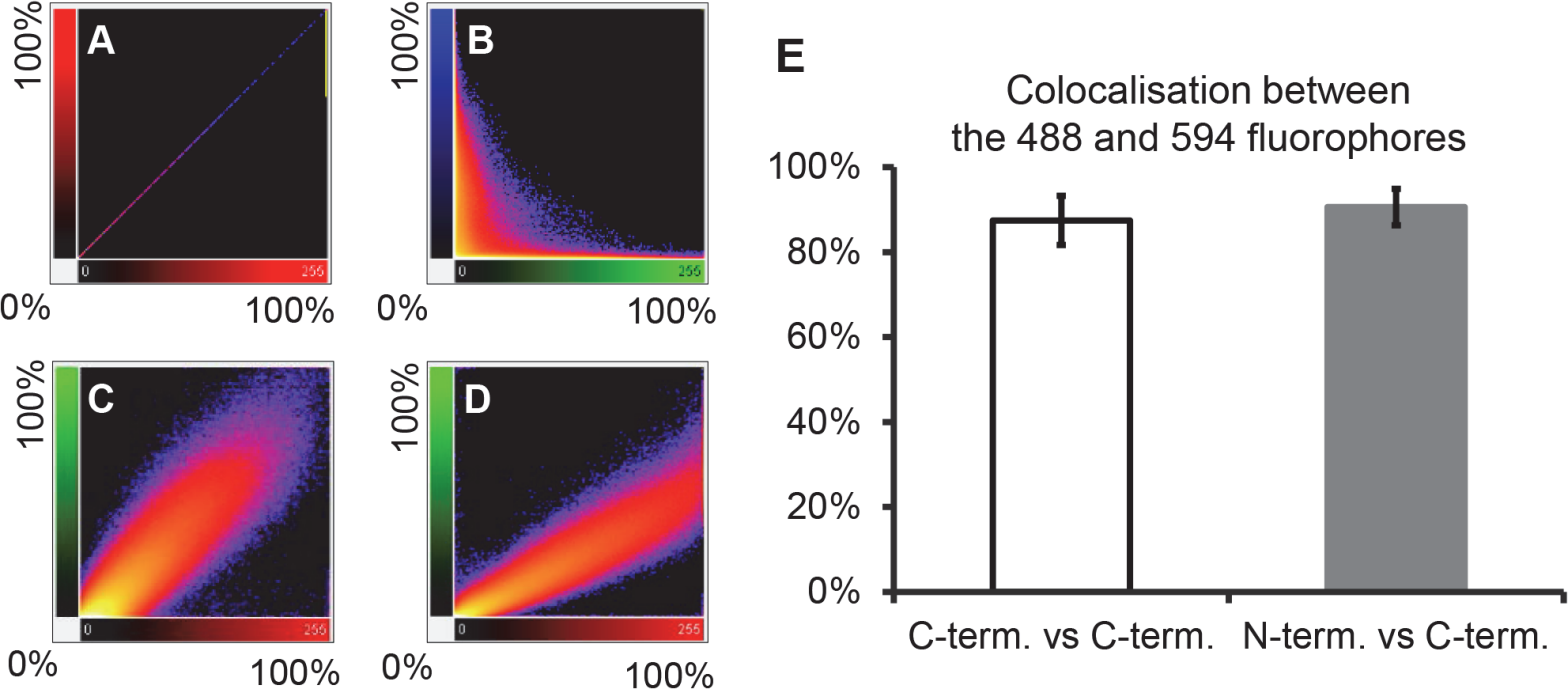
Quantitative analysis of colocalisation of the L protein N- and C-termini in infected cells. Three 3D images of infected cells, prepared as described for Fig 3 and each composed of eight focal slices, were analysed by the ImarisColoc function of the Imaris x64 version 7.4.2 software, using automatic threshold determination. **(A-D)** 2D plots of light intensity for each voxel in the 3D image for different pairs of channels for different pairs of antibodies: **(A)** plot of single channel against itself to illustrate theoretically perfect colocalisation; **(B)** plot of cytoplasmic L protein staining (green) vs nuclear DNA staining (blue) to illustrate perfect absence of colocalisation; **(C)** plot of actual perfect colocalisation, from staining infected cells with the same antibody (anti-L(CT)) labelled with two different fluorophores (Zenon 488 (green) or with Zenon 594 (red)); **(D)** plot of signal intensities given by anti-L(NT) and anti-L(CT). **(E)** Histogram showing average percentage of colocalisation (expressed as average “Pearson's coefficient in dataset volume”) between 488 and 594 signals from three analysed 3D images for each of control (infected cells stained only with anti-L(CT)) and experimental (infected cells co-stained with affinity purified anti-L(NT) and anti-L(CT)) samples. Error bars represent standard deviation.

To quantitatively analyse the colocalisation between the N-terminus and the C-terminus of the L protein, the series of images of focal planes through the thickness of an infected cell were analysed in a three-dimensional (3D) view using Imaris x64 version 7.4.2 software. For each sample, three independent 3D images were analysed by the ImarisColoc function of the software using the automatic threshold determination method [70]. For each analysed image, Imaris calculates the percentage colocalisation for the entire dataset volume of the Z-stack, building a “colocalised” channel (Fig 3D, 3H, 3L and 3P). The results for anti-N-terminus vs anti-C-terminus were compared to the percentage colocalisation obtained for three control datasets (Fig 4). Fig 4A–4D shows plots of the intensity of each channel for each voxel in the 3D images shown in Fig 3. A theoretically perfect colocalisation results in a linear correlation between the intensities of the two channels for every voxel (Fig 4A); in contrast, complete lack of colocalisation, as in the comparison of anti-L and the DNA stain DAPI (Fig 4B), results in complete lack of this linear correlation. In a real case of complete colocalisation (anti-L(C-terminus) vs itself), the correlation line is not perfect (Fig 4C), but shows a slight broadening reflecting experimental noise. It is nevertheless clear that the colocalisation between anti-L(N-terminus) and anti-L(C-terminus) in infected cells is as complete as the colocalisation observed for the control (Fig 4D), confirming that antibody to either end of the L protein is binding at essentially the same place (Fig 4E), suggesting that they are both part of the same molecule.

### Distribution of the N protein and the L protein in NSDV-infected cells

Using the specific anti-N and anti-L antibodies, we investigated the distribution of the N and L proteins in sheep epithelial cells (PO cells) and Vero cells. Both proteins were found in the cytoplasm of these infected cells and showed similar reticular staining patterns in both cell types (Fig 1B). Vero cells were chosen for further experiments since our pathogenic isolate of NSDV replicated better in these cells; in addition, many of Vero cells’ cellular components can be detected by widely available anti-human antibodies.

**Fig 5:**
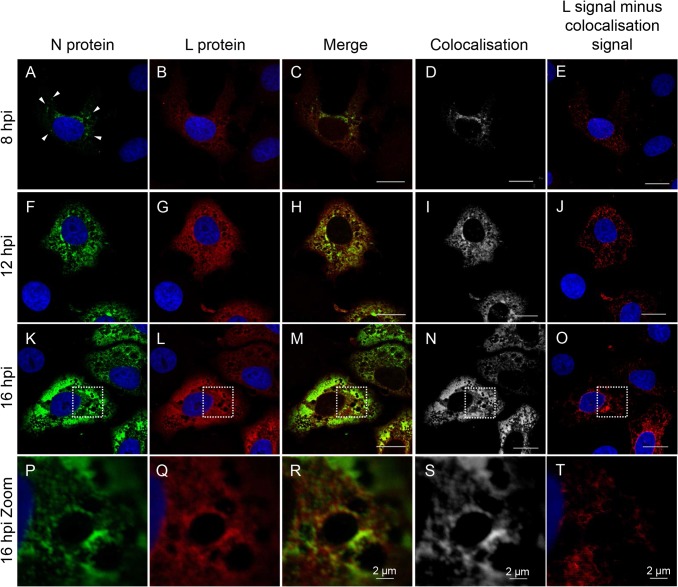
Distribution of the N and L proteins in NSDV-infected cells. Vero cells were infected with the NSDVi isolate at a MOI of 1 TCID_50_. After 8, 12 or 16 h cells were fixed with 4% PFA followed by ice cold methanol and were stained sequentially with affinity purified anti-L(CT) antibodies, Zenon AlexaFluor 594 (red) (Fab:antibody ratio 3.5) and pre-made complex of affinity purified rabbit anti-N antibodies with Zenon AlexaFluor 488 (green) (Fab:antibody ratio 3). Nuclei were counterstained using DAPI (blue). Representative focal planes from Z-stack series are shown. The ImarisColoc function of the Imaris x64 version 7.4.2 software was used to generate a colocalisation channel (D, I, N, S) for each time post infection using images composed of 6 (P-R), 10 (A-C) or 14 (F-H and K-M) focal planes through the thickness of an infected cell. To highlight areas where the L protein is present in the absence of the N protein, the colocalisation channel was subtracted from the L channel (red) (E, J, O, T). Bars correspond to 20 μm unless otherwise indicated. Dashed white boxes in (K-O) indicate the area shown enlarged in (P-T). Arrowheads in A highlight the punctate distribution of the N protein at 8 hpi.

Although the N protein and the L protein were observed to show similar staining patterns in the infected cells, it appeared that the distributions were not entirely identical (Fig 1B). To study the distribution of the N and L proteins in the same cell, affinity purified anti-N and anti-L antisera were directly labelled using the Zenon system, as described above, and the two antibodies were used to stain cells at different times post infection. Once again we took series of images (Z-stacks) for each infected cell using sequential scanning on a confocal microscope, and generated 3D immunofluorescence images which were used to quantify the colocalisation between the N and L proteins. As previously, the colocalisation data were normalized to the average percentage of colocalisation obtained for control images in which the infected cells were labelled with preparations of a single antibody labelled independently with the two fluorophores. This analysis allowed us to determine regions of colocalisation and to separate the signal from these regions from any remaining N or L signal. This analysis showed that the N and L proteins do not fully colocalise (Fig 5), particularly at early stages of infection. At 8 hpi, the N protein showed a punctate distribution throughout the cytoplasm with accumulation in the perinuclear area (Fig 5A), while the L protein was more evenly and widely distributed through the cytoplasm (Fig 5B), as well as accumulating in the perinuclear area, where it colocalised with the N protein (Fig 5B–5D). It was clear that, at this stage of the infection, the L protein also occupied significant areas of the cell in the absence of the N protein (Fig 5B–5E).

**Fig 6:**
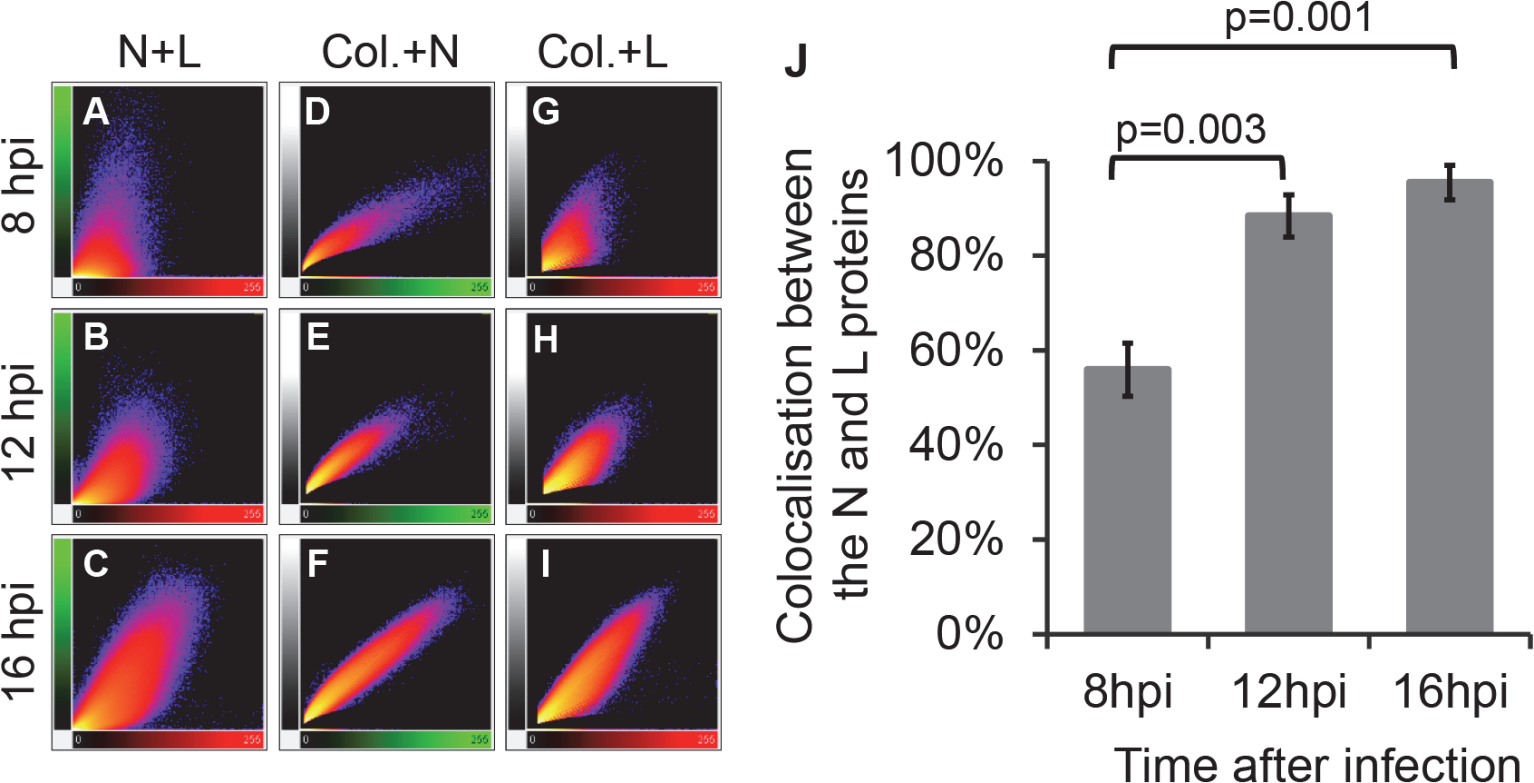
Quantitative analysis of colocalisation of the N and L proteins in infected cells. For each time point post infection, three 3D images, prepared as described for Fig 5 and each composed of 10 (8hpi) or 14 (12 and 16 hpi) focal slices, were analysed by the ImarisColoc function of the Imaris x64 version 7.4.2 software, using automatic threshold determination. (**A-I)** 2D plots of channel intensity in each voxel of the 3D images. **(A-C)** Plots showing the spatial correlation (colocalisation) of the N (green signal) and L (red signal) proteins over time (N+L); **(D-F)** plots showing the correlation between the total N-specific signal and the signal in the N-L colocalisation channel calculated by Imaris (white) over time (Col.+N); **(G-I)** corresponding plots of the correlation between the total L-specific signal and the signal in the N-L colocalisation channel (Col.+L). **(J)** Histogram showing normalised average percentage of colocalisation analysed for three different 3D images for each time point (expressed as average “Pearson's coefficient in dataset volume” between the N and L signal divided by average “Pearson's coefficient in dataset volume” of the positive control where infected cells were stained only with anti-L(CT), labelled independently with both fluorophores). Error bars represent standard deviation; p-values are for statistical comparison of colocalisation at 8 hpi compared to 12 and 16 hpi.

As the infection progressed and more viral protein was synthesised, the N protein became distributed more widely through the cytoplasm, covering the area already occupied by the L protein (Fig 5F–5I, 5K–5N and 5P–5S). However, even at 16 hpi, when a large part of the cytoplasm was occupied by the N protein, there were areas of the cell where only the polymerase was present (Fig 5O and 5T). A quantitative analysis of the amount of colocalisation confirmed these observations. The 2D plots of the intensities of the two fluorophores for each voxel show the changes in colocalisation between the N and L proteins over time (Fig 6). At early stages of infection there was only a little correlation between the red channel (L) and the green channel (N) (Fig 6A), which became more linear at later time points (Fig 6B–6C). Comparison of the relationships between the N or L proteins and the region of colocalisation (white; built using Imaris software) showed that the N protein had a linear relationship to the colocalisation data at all time points (Fig 6D–6F) while the L protein signal became more aligned with the N-L colocalisation channel over time (Fig 6G–6I), showing that the N protein is essentially only found where both viral proteins colocalise, while the L protein is present also in parts of the cell were the N protein is absent. At the early stages of infection, colocalisation between these two proteins was only 56%; colocalisation increased over the time, reaching >95% by 16 hpi (Fig 6J).

### Distribution of NSDV in tissues from experimentally infected animals

Very little is yet known about the pathology of the haemorrhagic gastroenteritis caused by NSDV; even the major organs and cellular targets are unknown. To learn more about the pathogenicity of NSD, we used the affinity-purified anti-N antibodies to detect NSDV in cryosections prepared from various tissues collected post-mortem from experimentally inoculated sheep which developed a haemorrhagic gastroenteritis after NSDVi infection, as previously described [18]. Parallel sections taken from samples of the same tissues removed from healthy sheep were used as controls.

**Fig 7:**
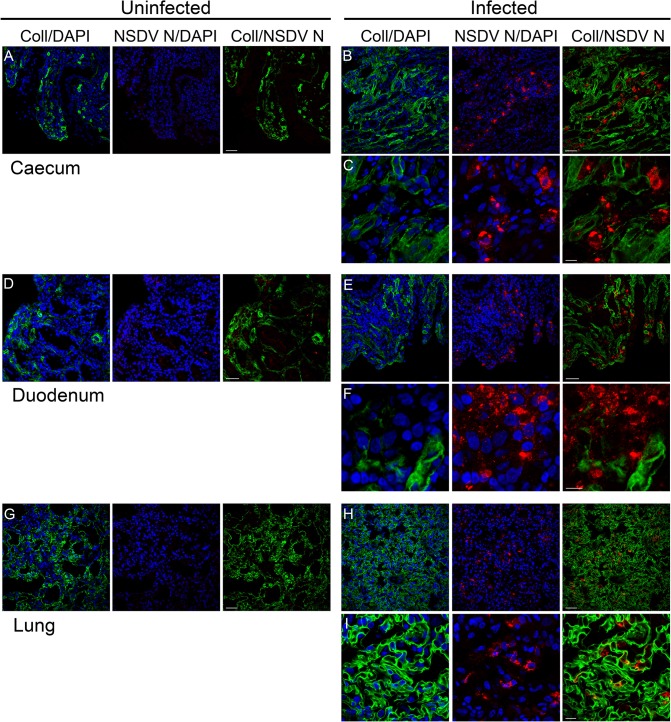
NSDV N protein distribution in caecum, duodenum and lung of infected sheep. Tissue samples were taken post-mortem from animals infected with the NSDVi isolate in a study previously described [18], or from healthy animals that were not subject to any experimental procedures. Cryosections were prepared and sections were fixed and stained as described in Methods, using mouse monoclonal anti-collagen IV antibody (Coll) and affinity-purified rabbit anti-NSDV N protein antibodies (NSDV N), followed by AlexaFluor 488 goat anti-mouse IgG (green) and AlexaFluor 568 goat anti-rabbit IgG (red). DAPI was used as a counterstain (blue). Scale bars indicate 40 μm (A, B, D, E, G, H) or 10 μm (C, F, I).

**Fig 8:**
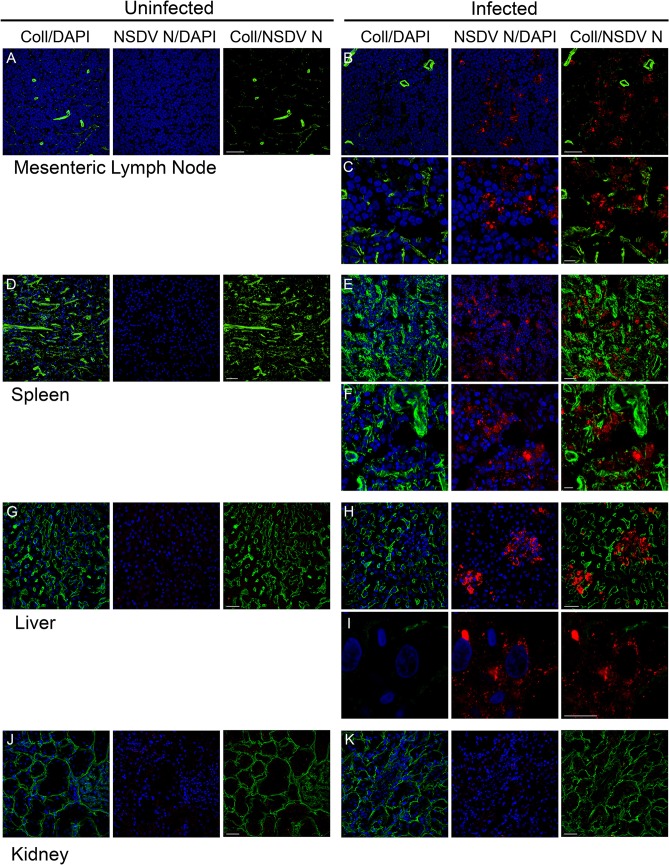
NSDV N protein distribution in lymph node, spleen, liver and kidney of infected sheep. Cryosections were prepared and stained as described for Fig 7. Scale bars indicate 40 μm (A, B, D, E, G, H, J, K) or 10 μm (C, F, I).

We detected the NSDV N protein in large amounts in the mucosal lining of the small and large intestine (duodenum and caecum), and the lungs (Fig 7), and in smaller foci in the liver, mesenteric lymph nodes (MLN), and spleen (Fig 8). No virus was found in kidney (Fig 8D) or aorta (not shown).

**Fig 9:**
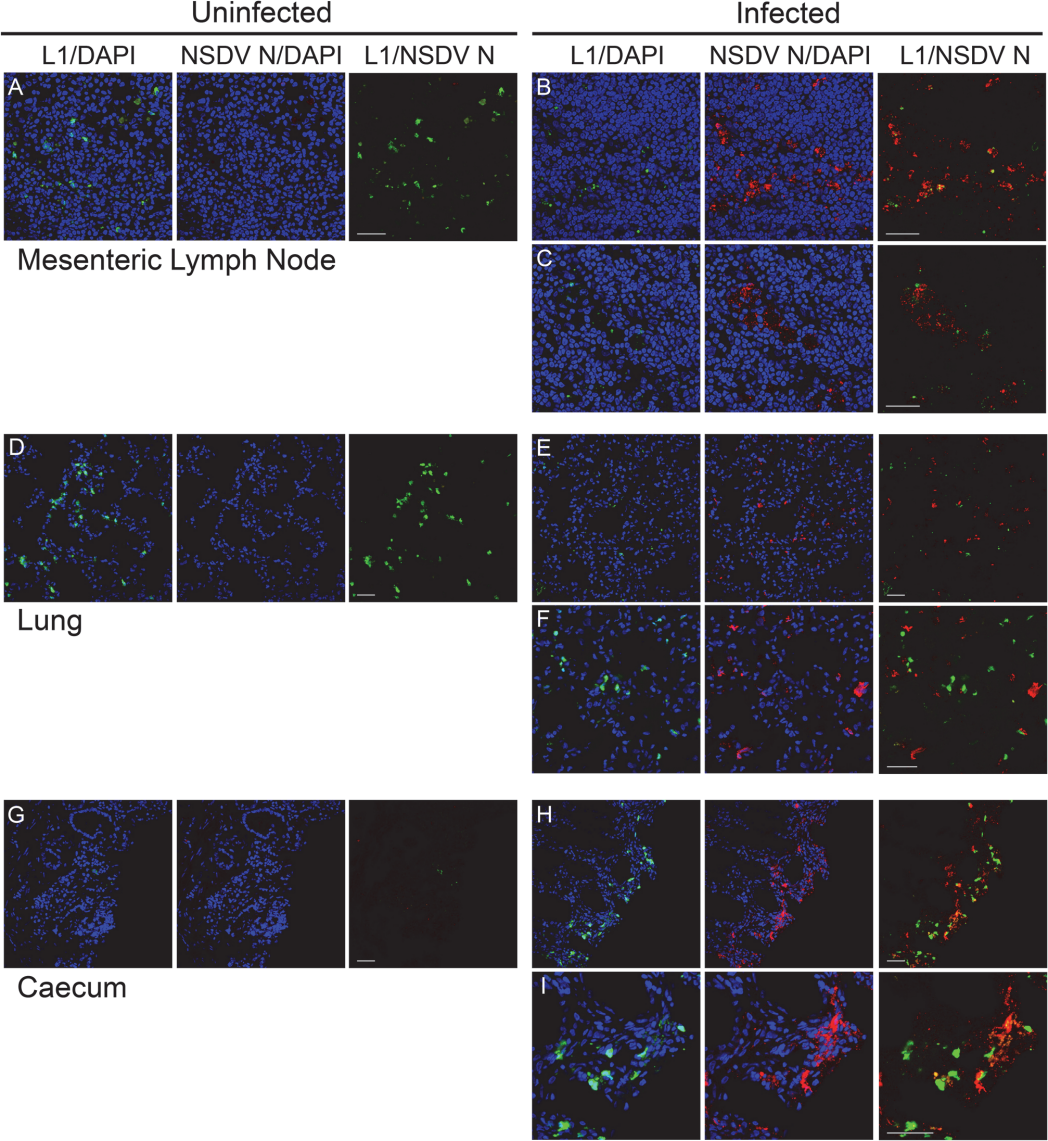
Effect of NSDV infection on distribution of macrophages/monocytes in experimentally inoculated sheep. Cryosections were prepared as described for Fig 7 and stained with mouse monoclonal anti-calprotectin/L1 antibody (L1) and affinity-purified rabbit anti-NSDV N protein antibodies (NSDV N), followed by Alexa Fluor 488 goat anti-mouse IgG (green) and Alexa Fluor 568 goat anti-rabbit IgG (red). DAPI was used as a counterstain (blue). Scale bars indicate 40 μm.

Although specific markers for well characterised cell type-specific antigens are not as common for sheep as they are for human and mouse, we co-stained the cryosections with antibodies against the viral N protein and a number of host cell proteins to try to identify the specific cellular target(s) of NSDV during natural infection. No correlation was seen between the staining for the N protein and the distribution of CD2 (T cells and NK cells), CD45 (general leukocyte marker), CD31 (endothelial cells marker) or cytokeratin (epithelial cells). Staining with antibody to calprotectin/L1, a general macrophage/monocyte marker, showed a noticeable effect of infection on these cells, particularly in the lung and the intestinal mucosa. In the lung there were many fewer L1^+^ cells in the infected animal’s tissue when compared to tissues from uninfected animals (Fig 9), although some staining of what appeared to be cell debris was observed. In contrast, in the caecum, there were many more L1^+^ cells in the wall of the gut in infected animals, while almost none were observed in the tissue of the uninfected animal (Fig 9). Additionally, L1-staining debris was often associated with the NSDV N protein (Fig 9), suggesting that macrophages/monocytes could be one of the cellular targets of the virus during natural infection.

## Discussion

Preparation of the affinity purified antibodies against the N- and the C-terminus of the NSDV L protein showed that, despite the presence of the OTU-like domain (a papain-like cysteine protease), the L protein does not appear to be (auto)proteolytically cleaved in infected cells, and behaves as a single, multifunctional protein. The same results were obtained in Vero and BHK-21 clone 13 cells infected with either the pathogenic (NSDVi) or the cell-culture adapted (NSDVu) isolate, which strengthens the biological relevance of this finding. It is of course possible that sections of both N- and C-termini are removed and preferentially rapidly degraded, leaving a core protein which would not be recognised by either of the anti-L antisera used in this paper. However, this would require an essentially simultaneous double cleavage of a kind not previously observed in negative strand RNA viruses. Our results are in agreement with studies investigating a plasmid-expressed CCHFV L protein where Western blot analysis of cells expressing the L-V5 fusion protein showed detection of only a full length CCHFV L-V5 protein [52]. Since the L protein of NSDV appeared to be very unstable in protein lysate samples, it is possible that additional, smaller, V5 tag-containing products observed by Bergeron and colleagues (2010) could be the result of unspecific degradation of the L-V5 protein. While inactivation of the OTU domain in BlScV results in a non-infectious virus [65], inactivation of the OTU domain in the CCHFV L protein has no effect on the polymerase activity, indicating that the CCHFV L protein folds correctly in the absence of an active OTU domain [52]; this further increases the likelihood that, just like the NSDV L protein, the CCHFV L protein remains as a single multifunctional protein.

The multifunctional nature of the NSDV L protein is further highlighted by the fact that the L and N proteins do not fully colocalise in infected cells. For most negative-sense RNA viruses, the N and L proteins act together to transcribe and encapsidate the viral vRNA and cRNA, and are normally found to interact together and therefore colocalise (e.g. the L protein of paramyxoviruses [73] or Ebola virus (EBOV; family *Filoviridae*) [74, 75], and PA-PB1-PB2 polymerase subunits of influenza virus (family *Orthomyxoviridae*) [76] all appear to localise only with areas of viral replication). This seems to be the case also for other bunyaviruses; the L protein of Bunyamwera virus (BUNV; genus *Orthobunyavirus*) and the L protein of Rift Valley fever virus (RVFV, genus *Phlebovirus*) showed high degrees of colocalisation with their respective N proteins in infected cells [77, 78]. However the L protein of NSDV, in addition to colocalising with the N protein, is also present in areas of the cytoplasm where the N protein is absent. Studies carried out in our laboratory showed that NSDV delays induction of antiviral responses, where the OTU-domain of the L protein takes part in the inhibition of IFNβ induction, and INFα or INFγ-induced transcription [60]. As the NSDV L protein appears to remain uncleaved, it probably distributes throughout the cytoplasm already from early stages of infection to block antiviral responses, giving the virus a replication window with no antiviral state. This also might be true for CCHFV, the OTU domain of which also appears to be involved in the delay of innate immune responses [61, 79]. The polymerase of hepatitis B virus (HBV; a DNA virus from the genus *Orthohepadnavirus*, family *Hepadnaviridae*) which also takes part in inhibition of innate antiviral responses [80, 81], similarly to the NSDV L protein, shows a widespread cytoplasmic distribution [82]. As yet, it is unknown whether the NSDV L protein which is not colocalised with the N protein is specifically associated with any cellular factors in infected cells.

Until now, from all genera of the family *Bunyaviridae*, only the L protein of nairoviruses has been described to act as a specific inhibitor of INF induction/action. It is possible that, since nairoviruses lack an accessory protein antagonistic to antiviral response (e.g. the NSs protein of orthobunyaviruses) [83, 84], the nairoviral L protein evolved to act as a multifunctional protein. Considering the fact that, apart from the OTU-domain, the L protein of nairoviruses contains additional domains which have not been found in other bunyaviruses e.g. predicted topoisomerase-like domain, zinc-finger domain and leucine zipper motif, all of which roles still remain unknown [44, 56], the L proteins might have other, still unidentified, functions in infected cells.

Distribution of the NSDV N protein in infected cells resembles that observed for CCHFV: the N protein of both viruses showed a punctate staining throughout the cytoplasm, then, still at the early stages of the infection, it relocated to a perinuclear area, showing punctate and filamentous staining around the Golgi, after which it further accumulated in the perinuclear area to finally build up in the entire cytoplasm at the late stages of infection [67, 79, 85, 86]. Accumulation of the N protein in the perinuclear area, in close proximity to the Golgi apparatus, would be expected for nairoviruses which, like most of bunyaviruses, assemble and bud into the Golgi where the N/RNP most probably interacts with the cytoplasmic tail of the Gn glycoprotein [50, 87–90].

Despite the fact that NSDV was identified over a century ago, the cell types or even organs targeted by the virus have been unknown. Using the antibodies against the N protein of NSDV allowed us to identify in which tissues/organs NSDV replicates in a natural host. The tissue sections from the experimentally infected sheep showed viral proteins present in cells in the mucosal layer of the gut (duodenum and caecum), as well as in the spleen, lung, liver and MLN; monocytes and/or macrophages appear to be targeted by the virus, but this requires confirmation. The lack of association of the NSDV marker with markers of endothelial and epithelial cells in the tissue sections from infected sheep, which correlates with poor growth of the pathogenic NSDV isolate in cultured epithelial and endothelial cells [18], suggests that epithelial and endothelial cells probably are not targeted by the virus.

Vascular damage, which is observed during viral haemorrhagic fevers (VHFs), can be caused directly by viral infection of endothelial cells or indirectly by activated inflammatory responses (reviewed in [91]); it is possible that during NSDV infection endothelial dysfunction, presenting as haemorrhage of the gum, gastrointestinal tract, spleen and heart [6, 18], is due to over-activated immunity rather that direct infection of endothelial cells, as has been proposed for other haemorrhagic viruses such as EBOV or DENV [92–99]. Replication of NSDV and CCHFV induce similar inflammatory responses in their respective hosts, which manifest predominantly by increased levels of interleukin (IL)-6, tumour necrosis factor (TNF)-α, IL-8, IL-10 and IFNγ [18, 100, 101]. Interestingly, CCHFV-infected monocyte-derived dendritic cells and macrophages have been shown to secrete these cytokines [102, 103] and CCHFV has been found to target mononuclear phagocytes during natural infection [104]. Similarly to CCHF patients, leukopenia has been observed in NSDV infected animals [18, 100].

Taken together above, it is probable that NSDV targets monocytes and/or macrophages during natural infection. The recruitment of these cells to the gut mucosa during infection, leading to a local increase in available host cells for the virus, may be a cause of the particular pathogenic effects of the virus on the gut.

## Supporting Information

S1 Fig Detection of viral proteins by immunofluorescence using generated rabbit antisera. Vero cells were infected with the NSDVi isolate at a MOI of 0.3 TCID_50_ or left uninfected. After 16 h, cells were fixed with 3% PFA, followed by ice cold methanol and viral proteins were immunolabelled using sera raised against the NSDV N, the C-terminus (C-term.) of the L protein or the N-terminus (N-term.) of the L protein followed by AlexaFluor-568 goat anti-rabbit IgG (red). DAPI was used as a counterstain (blue). Bars correspond to 40 μm.
(TIFF)

S2 Fig Validation of the Zenon IgG labelling system. Vero cells were infected with the NSDVi isolate at a MOI of 0.3 TCID_50_. After 16 h, cells were fixed in 4% PFA, followed by ice cold methanol. **(A, B)**: Cells were stained with rabbit antiserum against the C-terminus of the L protein, washed, stained with Zenon AlexaFluor 594 (red) rabbit IgG labelling reagent (400 ng of Fab in 20 μl) and washed again. Cells were then incubated with a pre-made labelling mix containing pre-immune serum from the rabbit that produced the anti-N antiserum coupled with Zenon AlexaFluor 488 (green) rabbit IgG labelling reagent (400 ng of Fab in 20 μl). **(C, D)**: Cells were sequentially incubated with pre-immune serum from the rabbit that produced the antiserum against the C-terminus of the L protein, Zenon AlexaFluor 594 (red) rabbit IgG labelling reagent (400 ng of Fab in 20 μl), and a pre-made labelling mix containing anti-N antiserum mixed with Zenon AlexaFluor 488 (green) rabbit IgG labelling reagent (400 ng of Fab in 20 μl), with extensive washing between each reagent. This was followed by a further series of washes and fixing with 4% PFA. Nuclei were counterstained using DAPI (blue). Bars correspond to 40 μm.
(TIFF)
