# Supplementary figures and images for: Antibodies to the Core Proteins of Nairobi Sheep Disease Virus/Ganjam Virus Reveal Details of the Distribution of the Proteins in Infected Cells and Tissues
Source: PLoS One. 2015 Apr 23;10(4):e0124966. doi: 10.1371/journal.pone.0124966 (PMC4407892; doi:10.1371/journal.pone.0124966)

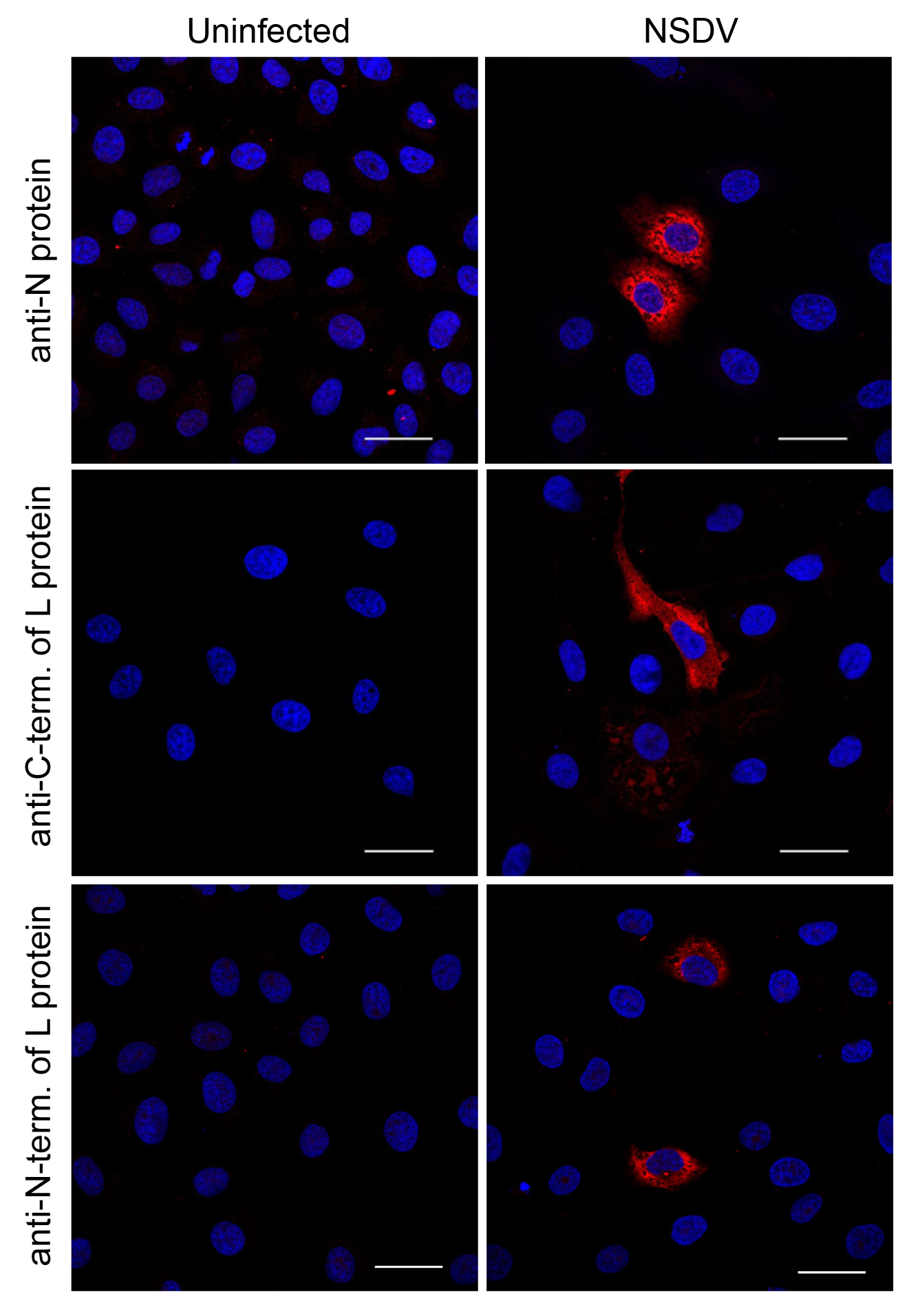

Supplement: S1 Fig — Vero cells were infected with the NSDVi isolate at a MOI of 0.3 TCID50 or left uninfected. After 16 h, cells were fixed with 3% PFA, followed by ice cold methanol and viral proteins were immunolabelled using sera raised against the NSDV N, the C-terminus (C-term.) of the L protein or the N-terminus (N-term.) of the L protein followed by AlexaFluor-568 goat anti-rabbit IgG (red). DAPI was used as a counterstain (blue). Bars correspond to 40 μm. (TIFF) [file pone.0124966.s001.tiff]

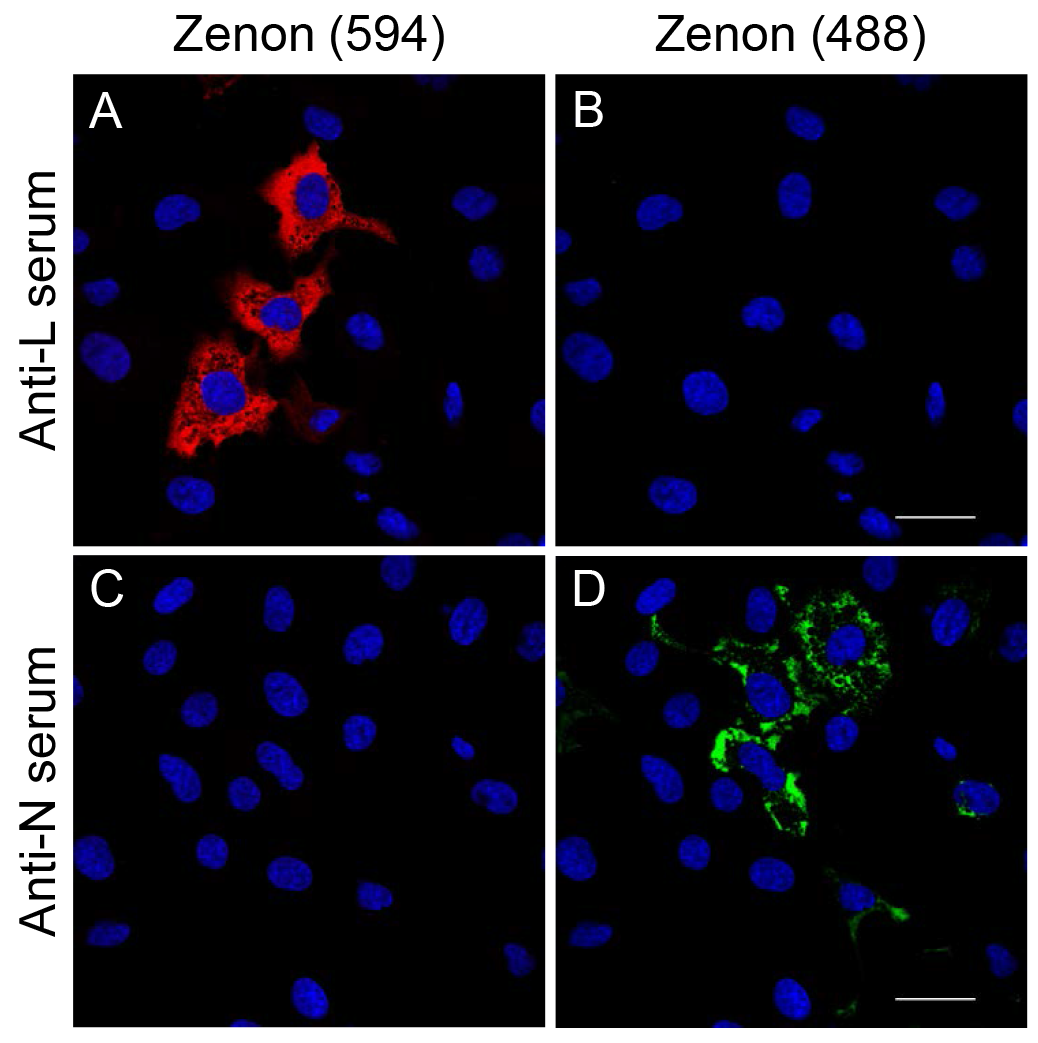

Supplement: S2 Fig — Vero cells were infected with the NSDVi isolate at a MOI of 0.3 TCID50. After 16 h, cells were fixed in 4% PFA, followed by ice cold methanol. (A, B): Cells were stained with rabbit antiserum against the C-terminus of the L protein, washed, stained with Zenon AlexaFluor 594 (red) rabbit IgG labelling reagent (400 ng of Fab in 20 μl) and washed again. Cells were then incubated with a pre-made labelling mix containing pre-immune serum from the rabbit that produced the anti-N antiserum coupled with Zenon AlexaFluor 488 (green) rabbit IgG labelling reagent (400 ng of Fab in 20 μl). (C, D): Cells were sequentially incubated with pre-immune serum from the rabbit that produced the antiserum against the C-terminus of the L protein, Zenon AlexaFluor 594 (red) rabbit IgG labelling reagent (400 ng of Fab in 20 μl), and a pre-made labelling mix containing anti-N antiserum mixed with Zenon AlexaFluor 488 (green) rabbit IgG labelling reagent (400 ng of Fab in 20 μl), with extensive washing between each reagent. This was followed by a further series of washes and fixing with 4% PFA. Nuclei were counterstained using DAPI (blue). Bars correspond to 40 μm. (TIFF) [file pone.0124966.s002.tiff]
